# Elderly Subjects Have a Delayed Antibody Response and Prolonged Viraemia following Yellow Fever Vaccination: A Prospective Controlled Cohort Study

**DOI:** 10.1371/journal.pone.0027753

**Published:** 2011-12-07

**Authors:** Anna H. Roukens, Darius Soonawala, Simone A. Joosten, Adriëtte W. de Visser, Xiaohong Jiang, Kees Dirksen, Marjolein de Gruijter, Jaap T. van Dissel, Peter J. Bredenbeek, Leo G. Visser

**Affiliations:** 1 Department of Infectious Diseases, Leiden University Medical Center, Leiden, The Netherlands; 2 Department of Medical Microbiology, Leiden University Medical Center, Leiden, The Netherlands; 3 Municipal Health Center Hollands Midden, The Hague, The Netherlands; 4 Municipal Health Center Leiden, Leiden, The Netherlands; Tulane School of Public Health and Tropical Medicine, United States of America

## Abstract

**Background:**

Yellow fever vaccination (YF-17D) can cause serious adverse events (SAEs). The mechanism of these SAEs is poorly understood. Older age has been identified as a risk factor. We tested the hypothesis that the humoral immune response to yellow fever vaccine develops more slowly in elderly than in younger subjects.

**Method:**

We vaccinated young volunteers (18–28 yrs, N = 30) and elderly travelers (60–81 yrs, N = 28) with YF-17D and measured their neutralizing antibody titers and plasma YF-17D RNA copy numbers before vaccination and 3, 5, 10, 14 and 28 days after vaccination.

**Results:**

Ten days after vaccination seroprotection was attained by 77% (23/30) of the young participants and by 50% (14/28) of the elderly participants (p = 0.03). Accordingly, the Geometric Mean Titer of younger participants was higher than the GMT of the elderly participants. At day 10 the difference was +2.9 IU/ml (95% CI 1.8–4.7, *p* = 0.00004) and at day 14 +1.8 IU/ml (95% CI 1.1–2.9, *p* = 0.02, using a mixed linear model. Viraemia was more common in the elderly (86%, 24/28) than in the younger participants (60%, 14/30) (p = 0.03) with higher YF-17D RNA copy numbers in the elderly participants.

**Conclusions:**

We found that elderly subjects had a delayed antibody response and higher viraemia levels after yellow fever primovaccination. We postulate that with older age, a weaker immune response to yellow fever vaccine allows the attenuated virus to cause higher viraemia levels which may increase the risk of developing SAEs. This may be one piece in the puzzle of the pathophysiology of YEL-AVD.

**Trial Registration:**

Trialregitser.nl NTR1040

## Introduction

The live attenuated 17D yellow fever vaccine is regarded as one of the safest and most effective vaccines [1]. However, in immunocompromized individuals yellow fever vaccination can cause fatal adverse events [2], [3]. A hampered immune response could allow the vaccine virus to replicate unrestrictedly, leading to vaccine-associated disease that resembles wild type yellow fever (yellow fever vaccine associated viscerotropic disease, YEL-AVD). YEL-AVD is fatal in 50% of cases [4]. In the last decade, a series of these serious and sometimes fatal adverse events following yellow fever vaccination has been reported [5]–[11]. The risk of YEL-AVD is increased for those with a history of thymectomy [12], male gender [13] and with increasing age. For vaccinees of 60–69 years this risk is estimated to be 1∶100.000 doses and for vaccinees of ≥70 years it is 2.3–3.2∶100.000, which is approximately a 4 and 11 fold higher risk than the risk for young adults [13], [14]. The higher risk of YEL-AVD in elderly travelers has resulted in a more restrictive policy towards vaccinating travelers of 60 years and older, also advised by the World Health Organisation and Centers for Disease Control en Prevention [15]–[18]. In this group the risk of serious adverse events following vaccination is weighed against the risk of infection, using disease surveillance data of the WHO and reports of yellow fever outbreaks.

The biological mechanism for the association between adverse events and older age has not yet been elucidated [4]. Both innate and adaptive immune responses wane with increasing age [19]. This may allow the attenuated vaccine virus more time to replicate and cause adverse events in elderly subjects. In this study we focused on humoral immunity, as this is considered to confer protective immunity against yellow fever. We tested the hypothesis that the adaptive immune response to yellow fever vaccine develops more slowly in elderly than in young subjects.

## Methods

The protocol for this trial and supporting checklist are available as supporting information; see Checklist S1 and Protocol S1.

### Ethics statement

The protocol and consent forms were approved by the Dutch Central Committee of Human Research (CCMO) and by the Medical Ethical Committee of the Leiden University Medical Center (LUMC) in the Netherlands. The trial was registered under NTR1040 and ISRCTN42180653, (http://irsctn.org). Written informed consent was obtained from each participant prior to inclusion.

### Objectives

This study was conducted to determine whether the adaptive immune response to yellow fever vaccine is slower to develop in persons of 60 years or older compared with persons aged 18 to 40 years. Primary outcomes were the humoral response to yellow fever vaccination, measured by Plaque Reduction Neutralization Test (PRNT), and Yellow Fever 17D (YF-17D) viraemia after vaccination, which was quantified by real time PCR (qRT-PCR). Secondary outcomes were adverse events.

### Study design and Participants

In this prospective controlled cohort study, participants were recruited at the Travel Clinic of the Leiden University Medical Center (LUMC), and Municipal Health Centers of Leiden and The Hague, the Netherlands. Healthy volunteers aged between 18 and 40 years and eligible for inclusion into the control group were invited to participate. Participants in the control group were not necessarily planning to travel to a yellow fever endemic area. The study group consisted of healthy travelers aged 60 years or above, who had an indication for yellow fever vaccination based on their travel destination (National Coordination Center for Travelers' Health, LCR) [20]. Individuals who had previously received yellow fever vaccine or who had a compromised immunity due to underlying illness or immunosuppressive medication and those who were pregnant were excluded. The study was carried out between April 2008 and April 2009. Vaccinations were administered at the Travel Clinic of the LUMC by AR. The trial ended because the number of inclusions was met.

### Yellow fever vaccine

The live, attenuated, 17D vaccine used in this study was manufactured on embryonated chicken eggs according to WHO regulations and stored according to manufacturer's guidelines. All administered vaccines originated from the same vaccine lot (Stamaril, Lot no B5355, Sanofi Pasteur, France). The vaccine was administered subcutaneously in the deltoid region of the right arm.

### Data collection

At the time of inclusion, data on demographic characteristics of the participants were obtained. Blood samples for the determination of neutralizing antibodies (NA) and YF-17D viraemia were collected before (day 0), and 3, 5, 10, 14 and 28 days after vaccination. Participants were asked to document any injection site and systemic adverse events after vaccination in a three-week diary. Solicited symptoms were: erythema, pain and swelling at the site of injection, fever and myalgia. Non-solicited symptoms could also be reported.

### Constant virus – varying serum dilution Plaque Reduction Neutralization Test (PRNT)

The tests were carried out in 6-well plates (Corning Inc., USA) using a slightly modified technique described originally by De Madrid and Porterfield [21]. Briefly, approximately 6×10^5^ Vero cells/mL were seeded per well in 6-well plates and cultured to obtain a confluent monolayer. Coded sera were complement inactivated at 56°C for 1 hour. Pre-vaccination sera were tested in 1∶16 dilution, to which 100 plaque forming units (PFU) of 17D-YF were added. Post vaccination sera were tested in two-fold dilutions starting from 1∶4 to 1∶1024. One hundred PFU of YF-17D virus were added to each serum dilution. All test sera were assayed in duplicate. After 1 hour incubation on ice, the mixtures of virus and serum were added to the Vero cell monolayers and incubated for 1 hour at 37°C. An overlay of 2×DMEM and 2% agarose was added. After 5 days of incubation at 37°C, the overlay was discarded and cell monolayers were stained with crystal violet. Plaques were counted by eye by a person who had no access to the sample code. Virus neutralization (VN) was calculated for each serum dilution (i) with the following formula: VN(i) = 100×1−(number of PFU in diluted post vaccination serum/number of PFU in pre-vaccination serum (in a 1∶16 dilution)). The serum dilution at which log_10_ neutralization index 0·7 (80% VN) occurred was taken as endpoint, as this corresponds to the World Health Organization (WHO) definition of protection [22]. A reference serum, obtained from the National Institute for Biological Standards and Control (http://www.nibsc.ac.uk/) was used for quantification of International Units per milliliter (IU/ml). In our hands a 0.7 log10 plaque reduction in 1∶10 diluted serum corresponds to a titer of 0.5 IU/ml [95%CI 0.3–0.8 IU/ml] [23]. Similar values have been found by others [24]. Geometrical mean titers (GMT) were compared between the two groups.

### Reverse Transcriptase-Polymerase Chain Reaction (RT-PCR)

Viral RNA was isolated from 200 µl plasma using a MagNa Pure LC Total Nucleic Acid Isolation Kit (Roche Molecular Diagnostics, Penzberg, Germany). cDNA was synthesized with 10 µl elute (200 µl total) in a professional ThermoCycler (Biometra, Germany), and quantitative reverse transcription-PCR (qRT-PCR) of YFV RNA was performed in a BioRad i-cycler IQ™ real-time PCR detection system (BioRad, Veenendaal, The Netherlands). The following YFV specific primers and probe were used [25]:

YFV-1 (forward) AATCGAGTTGCTAGGCAATAAACAC

YFV-2 (reverse) TCCCTGAGCTTTACGACCAGA

YFV-P (probe) FAM-ATCGTTGAGCGATTAGCAG-BHQ

FAM (6-carboxyfluorescein) was used as 5′-reporter dye and BHQ (Black Hole Quencher) as 3′-quencher dye. In order to quantify YFV RNA, log_10_ dilutions of in vitro transcribed RNA standards were included as standard curves. RNA virus levels were calculated with standard curves from Cycle threshold (Ct) values to compare viraemia in both groups quantitatively, and were expressed as IU/ml.

### Statistical methods

Power calculations were based on an expected 80% virus neutralization of 95% in the control group and 66% in the elderly group at day 14, based on previous observations at the Travel Clinic (unpublished data). With an α of 0·05 and β of 0·2, 26 participants per group were needed to confirm a significant difference under these assumed conditions. To take into account a possible attrition rate of 15%, 30 participants were included per group. We analyzed the between group difference in GMT over the four time points (day 5, 10, 14, 28) using a mixed linear model. This model takes into account that each subject had repeated measurements of the antibody titer over time. More specifically, a unique identification number for each subject was entered as a random effect in the model and separate variables for all time points and for the groups (elderly versus young) were entered as fixed effects. Antibody titers below the detection threshold were assigned an arbitrary value of 0.05 IU/ml, which is twofold lower than the lowest detectable titer (i.e. 0.2 IU/ml). Where appropriate, Chi-square tests were used, and Wilcoxon's test for non-parametrical distributed numerical data. Statistical analysis was performed using a computer-assisted software package (SPSS version 16.0, SPSS Inc., Chicago, IL).

## Results

### Population

We enrolled 60 participants, none of whom withdrew prematurely. In 2 elderly participants, 17D-YF neutralizing antibodies were already present at day 0. In retrospect, these participants remembered that they were vaccinated against yellow fever many years ago. These two individuals were excluded from further analysis. In both groups 70% were female and 30% had visited flavivirus endemic countries in the past. The median age of the younger participants was 21 years (interquartile range 20–22.5) and of the elderly was 66 years (interquartile range 65–69). Although we invited persons of 18 to 40 years of age for the control group, the oldest participant in this group was 28 years old. Therefore the control group is defined as age 18–30 years. We recorded the incidence of previous travel to countries that are endemic for flaviviruses because past infections with other flaviviruses can cause cross-neutralization in the YF PRNT.

### Neutralizing antibody response

**Figure 1:**
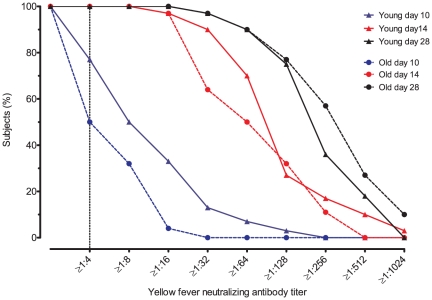
Neutralizing antibody response against YF-17D in young and elderly participants. Reverse cumulative distribution curves of yellow fever neutralizing antibody titers at 5, 10, 14 and 28 days after vaccination in 30 young and 28 elderly participants. Antibody titers were determined with Plaque Reduction Neutralization Tests and reflect the serum dilution at which 80% of virus was neutralized.

At day 3 and 5 after vaccination, no neutralizing antibodies were found in any of the participants. Ten days after vaccination seroprotection was attained by 77% (23/30) of the young participants and by 50% (14/28) of the elderly participants (p = 0.03, Chi-square test) (figure 1). The average GMT taken over the four time points after vaccination was higher in the group of young participants compared with the group of elderly participants. The average difference in GMT was +1.7 IU/ml (95% CI 1.2–2.4, *p* = 0.007). At day 10 the difference was +2.9 IU/ml (95% CI 1.8–4.7, *p* = 0.00004) and at day 14 +1.8 IU/ml (95% CI 1.1–2.9, *p* = 0.02). At day 28 the difference was no longer statistically significant (+1.5 IU/ml, 95%CI 0.9–2.4, *p* = 0.12). Female participants in the elderly group had a higher antibody response 10 days after vaccination (female vs. male 0.04 IU/ml (95% CI 0.01–0.15) vs. 0.002 IU/ml (95%CI 0.0005–0.01), *p* = 0.03). Such a difference between men and women was not seen in the group of young participants.

### Yellow fever vaccine virus RNA

YF-17D viraemia was measured by qRT-PCR at day 0, 3, 5, 10 and 14 (table 1). Viraemia was detected more often in elderly (24/28, 86%) than in young participants (18/30, 60%) (p = 0.04, Chi-square test). In addition, the elderly had higher viraemia levels detectable for longer periods and two had detectable viraemia at day 10, compared with none of the younger participants (table 1).

**Table 1:** YF-17D viraemia measured by qRT-PCR in the elderly group compared to young participants.

YF-17D viraemia		Young N = 30	Elderly N = 28	p-value
Day 0	Number positive (%)	0 (0)	0 (0)	-
Day 3	Number positive (%)	6 (20)	11 (39)	0.1
	IU/ml (95% CI)	1.4 (0.9–1.9)	2.9 (2.1–4.4)	0.04
Day 5	Number positive (%)	16 (53)	23 (82)	0.02
	IU/ml (95% CI)	4.8 (0–10.7)	20.8 (10.2–31.5)	0.07
Day 10	Number positive (%)	0 (0)	2 (7)	0.2
	IU/ml (95% CI)	-	1.00 (0.8–1.2)	-
Day 14	Number positive	0 (0)	0 (0)	-
1 time point positive (%)		14 (78)	12 (50)	0.02
2 sequential time points positive (%)		4 (22)	12 (50)	

YF-17D RNA virus levels were calculated with standard curves from Cycle threshold (Ct) values and were expressed as IU/ml. Comparison of number of participants positive for viraemia was calculated by Fisher's Exact test. Comparison of quantitative viraemia (only of participants who had measurable viraemia) was calculated with Student's t-test. IU = International Units, 95% CI = 95% Confidence Interval.

### Adverse events

Participants reported the occurrence and duration of adverse events after yellow fever vaccination in a 3-week diary (table 2). In younger participants vaccination evoked erythema at the site of inoculation more frequently and for a longer period than in the elderly participants. In both groups, viraemia peaked at day 5. In the group of elderly participants the mean viraemia level at day 5 was higher in those who experienced a systemic adverse event (fever and/or myalgia) than in those who did not (viraemia level 31.3 versus 11.5 IU/ml, 95% CI for the difference 0.4–40.0 IU/ml), p = 0.05). In the group of young participants mean viraemia levels did not differ significantly between those who did experience a systemic adverse event and those who did not (viraemia level 6.1 versus 3.9 IU/ml respectively).

**Table 2:** Solicited adverse events after primary and booster YF-17D vaccination.

Adverse event (AE)			Young N = 30	Elderly N = 28	p-value
Injection site AE	Any	Yes (%)	9 (30)	4 (14)	0.15
		Days to onset (range)	0 (0-2)	0.5 (0-6)	0.6
	Erythema	Yes (%)	8 (27)	2 (7)	0.05
		Days duration (range)	2.5 (1-8)	2 (1-3)	0.4
	Swelling	Yes (%)	3 (10)	1 (4)	0.3
		Days duration (range)	2 (1-5)	2 (-)	1.0
	Pain	Yes (%)	3 (10)	2 (7)	0.7
		Days duration (range)	1 (1-3)	2 (2-2)	0.5
Systemic AE	Any	Yes (%)	12 (40)	8 (29)	0.4
		Days to onset (range)	0.5 (0-4)	5 (1-6)	0.002
	Myalgia	Yes (%)	12 (40)	6 (21)	0.4
		Days to onset (range)	1 (0-6)	5 (1-6)	0.12
	Fever	Yes (%)	3 (10)	4 (14)	0.6
		Days to onset (range)	0 (0-4)	5 (5-6)	0.03

Safety of vaccination expressed in various parameters. Numbers of days are medians. Fever was defined as self-measured temperature above 38 degrees Celsius. P-values based on Chi-square test and Wilcoxon's test. AE = Adverse event.

## Discussion

The main finding of this study was that after primary vaccination with 17D YF vaccine, elderly persons (≥60 years) were slower to develop an antibody response and had higher viraemia levels than younger persons. Only half of the elderly vaccinees had protective antibody levels 10 days after vaccination compared with over three quarters of younger vaccinees. In addition, GMT of neutralizing antibodies were significantly lower at 10 and 14 days after vaccination. The difference was less pronounced and no longer statistically significant 28 days after vaccination. Besides showing higher levels of viraemia in elderly subjects, our data also suggest that the duration of viraemia is prolonged in these subjects as two elderly participants and none of the younger participants had detectable viraemia at day 10.

These results provide insight into the etiology of the increased susceptibility to YEL-AVD after yellow fever vaccination in old age. Immunosenescence leading to an impaired ability to clear the vaccine virus has been put forth as a possible explanation for the increased risk of YEL-AVD in elderly people [26]. However, in a retrospective study of two large 17D vaccine trials involving 4,532 subjects, neutralizing antibody responses at 30 days after vaccination were equivalent in younger and elderly subjects. Due to the retrospective nature of that study, early responses (i.e. <30 days after vaccination) could not be compared and were assumed to be equal in both groups. Our results show that this assumption needs to be modified, as we show that elderly vaccinees are slower to develop an antibody response than younger vaccinees. This cannot entirely explain higher age as a risk factor for YEL-AVD, as viraemia levels peak at day 5, before the development of neutralizing antibodies. The innate immune response is probably also an important factor influencing viral replication after vaccination, as suggested by Silva and colleagues [27]. We think that the higher viraemia levels in elderly subjects may be due to a weaker innate immune response. Such a hampered innate immune response together with a slower humoral response could allow the YF-17D virus to replicate more efficiently and for a longer period of time increasing the chance of YEL-AVD. In this respect it is interesting to note that the incidence of adverse events at the injection site was lower in elderly than in younger subjects. If reactions at the injection site are the result of immune activation, observing less injection site adverse events in elderly subjects could reflect a weaker or slower innate immune response in elderly persons. Similar observations were made in an earlier study of yellow fever vaccination in elderly subjects [26].

Beside immunosenescence in elderly subjects, other factors contributing to YEL-AVD have been postulated. For example, it has previously been suggested that the vaccine virus reverts or mutates to a more virulent form during replication in a vaccinated individual, but extensive genetic analyses of the viral strains extracted from patients with YEL-AVD do not provide evidence to support this hypothesis [4]. The possibility of host genetic susceptibility for developing YEL-AVD seems more plausible. Pulendran and colleagues found a heterozygous CCR5Δ32 mutation in a patient who suffered from YEL-AVD [28]. Since the prevalence of heterozygosity of the CCR5Δ32 mutation in the general population is 15% [29] and the occurrence of YEL-AVD among yellow fever vaccinees is significantly less [13], [14], additional host factors (e.g. immunosenescence) must also play a role in the development of YEL-AVD [30]. On the other hand, milder forms of YEL-AVD might occur more frequently, but might not be severe enough to be published, thus introducing publication bias. Supportive of the hypothesis of genetic susceptibility, other recently discovered genetic host factors, including complement protein C1qB and eukaryotic translation initiation factor 2 alpha kinase 4- (an orchestrator of the integrated stress response) predicted YF-17D CD8^+^ T cell responses with up to 90% accuracy and a B-cell growth factor, TNFRS17, predicted the neutralizing antibody response with up to 100% accuracy [31].

Although occurrence of YEL-AVD is very rare, fear of this adverse event could reduce utilization of yellow fever vaccine. An “International Laboratory Network for Yellow Fever Vaccine-Associated Adverse Events” has been established in 2008, to complement the USA and the European Yellow Fever Vaccine Safety Working Groups [32]. Its goal is to determine the pathogenesis of severe adverse events following yellow fever vaccination through systematic and coordinated laboratory evaluation of reported cases. A greater understanding of the pathogenesis of YEL-AVD may lead to new approaches to prevent this serious complication. One strategy may be to inject less vaccine virus in a more immunostimulant manner (e.g. intradermally) [33]. Alternatively, inactivated YF-17D vaccine could be used to prime the immune response which can be boosted later with live attenuated YF-17D. This strategy has been successfully used in mice, hamsters and cynomolgous monkeys [34], and more recently Monath en co-workers have demonstrated an adequate antibody response against yellow fever following inactivated yellow fever vaccine [35].

The findings of our study can have the following practical implication: in travelers of 60 years and older, it would be prudent to vaccinate against yellow fever at least 14 days instead of 10 days before departure to guarantee that all vaccinees have obtained protective antibody levels.

## Supporting Information

Protocol S1 **Trial Protocol.**
(DOCX)

Checklist S1 **CONSORT Checklist.**
(DOC)
