# Supplementary material for: Elderly Subjects Have a Delayed Antibody Response and Prolonged Viraemia following Yellow Fever Vaccination: A Prospective Controlled Cohort Study
Source: PLoS One. 2011 Dec 7;6(12):e27753. doi: 10.1371/journal.pone.0027753 (PMC3233541; doi:10.1371/journal.pone.0027753)
Supplement: Protocol S1 — Trial Protocol. (DOCX) [file pone.0027753.s001.docx]

**Onderzoeksprotocol**

**Titel** Onderzoek naar immuunrespons na gele koorts vaccinatie bij oudere (>60jaar) reizigers vergeleken met jongeren (18 t/m 40 jaar)

| **Protocol ID** | NL18398.000.07 |
| --- | --- |
| **Korte titel** | Gele koorts vaccinatie bij ouderen |
| **Versie** | 4 |
| **Datum** | 29 mei 2008 |
| **Coördinerende onderzoeker** | Drs. A. Roukens  Afd. Infectieziekten, C5-P, LUMC  Postbus 9600  2300 RC Leiden  Tel: 071-5261974 / 2613  Fax: 071-5266758  E-mail: A.H.E.Roukens@lumc.nl |
| **hoofdonderzoeker/uitvoerder** | - LUMC (drs. A. Roukens en dr. L. Visser)  Tel: 071-5261974 |
| **verrichter/opdrachtgever** | Afdeling Infectieziekten LUMC |
| **Onafhankelijke arts** | Dr. F. Kroon  Tel: 071-5262613 |
| **Laboratorium** | Laboratorium voor Infectieziekten, LUMC |

# Protocol handtekeningenformulier

| **Naam** | **Handtekening** | **Datum** |
| --- | --- | --- |
| **Niet-commercieel onderzoek**  **Hoofd van de afdeling:**  Prof. J.T. van Dissel  Hoogleraar afd. Infectieziekten LUMC |  |  |
| **Coördinerende onderzoeker**  Drs. A.H.E. Roukens,  AIO afd. Infectieziekten |  |  |

# Inhoudstabel

[Protocol handtekeningenformulier 2](#_Toc178569819)

[Inhoudstabel 3](#_Toc178569820)

[Samenvatting protocol 5](#_Toc178569821)

[1. Inleiding en rationale 6](#_Toc178569823)

[2. Vraagstellingen 7](#_Toc178569825)

[3. Doel 7](#_Toc178569826)

[4. Onderzoeksontwerp 7](#_Toc178569827)

[5. Onderzoekspopulatie 8](#_Toc178569828)

[5.1 Populatie 8](#_Toc178569829)

[5.2 Inclusiecriteria 8](#_Toc178569831)

[5.3 Exclusiecriteria 9](#_Toc178569832)

[5.4 Berekening van de populatiegrootte 9](#_Toc178569833)

[6. Behandeling van deelnemers 9](#_Toc178569834)

[6.1 Onderzoeksproduct 9](#_Toc178569835)

[6.2 Co-interventies 10](#_Toc178569836)

[7. Medicatie gebruikt in het onderzoek 10](#_Toc178569837)

[8. Onderzoeksmethoden 10](#_Toc178569838)

[8.1 Eindpunten 10](#_Toc178569839)

[8.1.1 Primaire eindpunten 10](#_Toc178569840)

[8.1.2 Secundaire eindpunten 11](#_Toc178569841)

[8.2 Studie procedures 11](#_Toc178569842)

[8.3. Terugtrekking uit het onderzoek 12](#_Toc178569843)

[9. Bijwerkingenrapportage 12](#_Toc178569844)

[9.1 Sectie 10 WMO event 12](#_Toc178569845)

[9.2 Bijwerkingen en ernstige bijwerkingen 12](#_Toc178569846)

[9.2.1 Suspected unexpected serious adverse reactions (SUSAR) 13](#_Toc178569847)

[9.2.2. Jaarlijkse bijwerkingen rapport 13](#_Toc178569848)

[9.3 Follow-up van bijwerkingen 13](#_Toc178569849)

[10. Statistische analyse 13](#_Toc178569850)

[11. Ethische overwegingen 14](#_Toc178569851)

[11.1 Regulation statement 14](#_Toc178569852)

[11.2 Recrutering en toestemming 14](#_Toc178569853)

[11.3 Verantwoording voor het onderzoek en groeps-gerelateerdheid 14](#_Toc178569855)

[11.4 Vergoeding 15](#_Toc178569857)

[11.5 Compensatie voor schade door deelname aan het onderzoek 15](#_Toc178569858)

[12. Administratie en publicatie 15](#_Toc178569859)

[12.1 Omgang en opslag medische- en onderzoeksgegevens 15](#_Toc178569860)

[12.2 Amendementen 16](#_Toc178569861)

[12.3 Jaarlijkse rapportage 16](#_Toc178569862)

[12.4 Rapportage van het einde van de studie 16](#_Toc178569863)

[12.5 Verslaglegging 16](#_Toc178569864)

[13. Referenties 16](#_Toc178569865)

[Bijlage 1 18](#_Toc178569866)

[Bijlage 2 19](#_Toc178569867)

[Bijlage 3 20](#_Toc178569868)

[Bijlage 4 21](#_Toc178569869)

# Samenvatting protocol

**Titel**  Onderzoek naar de immuunrespons na gele koorts vaccinatie bij oudere (>60 jaar) reizigers vergeleken met jongeren (18 t/m 40 jaar).

Het gele koorts vaccin is een levend verzwakt virusvaccin, dat bij een minder goed functionerend immuunsysteem de ziekte gele koorts kan veroorzaken. Enkele jaren geleden is bij reizigers ouder dan 60 jaar een verhoogde kans op gele koorts vaccinatie geassocieerde ziekte (YEL-AVD) geconstateerd op basis van enkele fatale gevallen. Het biologisch mechanisme van deze mogelijk verhoogde kans op zeer zeldzame bijwerkingen is niet bekend. Mogelijk komt de immuunrespons na vaccinatie bij ouderen langzamer op gang (bijvoorbeeld door de leeftijdsafhankelijke afname van naïeve lymfocyten of toegenomen aantal T regulatoire cellen), waardoor de gele koorts viremie langer aanhoudt en hoger is dan bij jongeren.

In een multicenter prospectieve cohortstudie wordt de immunologische respons op gele koorts vaccinatie bij reizigers van 60 jaar en ouder onderzocht aan de hand van de humorale en cellulaire immuniteit, en wordt de viremie na vaccinatie gemeten. Hiervoor wordt één keer gevaccineerd en zes keer bloed en urine afgenomen in een tijdsbestek van één maand.

De reizigers die voor deelname in aanmerking komen zijn diegenen die 60 jaar of ouder zijn, en op basis van hun reis een indicatie hebben voor gele koorts vaccinatie. Dat wil zeggen dat bij deze reizigers het risico op bijwerkingen na vaccinatie kleiner is dan het risico op de ziekte vanwege hun reisbestemming.

# De uitkomst van dit onderzoek kan een bijdrage leveren aan de opheldering van de mogelijk leeftijdsafhankelijke kinetiek van deze respons, en van de immuunrespons geïnduceerd door gele koorts vaccinatie.

**Titel** Onderzoek naar immuunrespons na gele koorts vaccinatie bij oudere (>60

jaar) reizigers vergeleken met jongeren (18 t/m 40 jaar)

# 1. Inleiding en rationale

Het gele koorts vaccin is een levend verzwakt virusvaccin, dat bij een minder goed functionerend immuunsysteem de ziekte gele koorts kan veroorzaken. Enkele jaren geleden is bij reizigers ouder dan 60 jaar een verhoogde kans op gele koorts vaccinatie geassocieerde ziekte (YEL-AVD) geconstateerd op basis van enkele fatale gevallen. (1;2) Dit heeft geleid tot een verscherpt beleid rond de indicatiestelling voor gele koorts vaccinatie van reizigers ouder dan 60 jaar. Indien deze personen naar een endemisch gebied reizen waar gele koorts nauwelijks of niet voorkomt, wordt afgezien van vaccinatie. In de overige gevallen wordt op grond van het Landelijk Coördinatiecentrum Reizigersadvisering (LCR) protocol gele koorts vaccin toegediend.

Het biologisch mechanisme van deze verhoogde kans op bijwerkingen is niet bekend. In een onderzoek waarbij de neutraliserende antistofrespons op twee verschillende gele koorts vaccins retrospectief bij ouderen t.o.v. jongeren bestudeerd werd, werd geen verschil gevonden in de antistoftiter 30 dagen na vaccinatie. (3) Echter, bij een nog niet gepubliceerd onderzoek uitgevoerd door de coördinerende onderzoeker op de afdeling Infectieziekten van het LUMC werd bij 2 van de 3 deelnemers ouder dan 60 jaar 14 dagen na vaccinatie een respons gemeten die niet beschermend was tegen gele koorts. Alle deelnemers die jonger waren dan 60 jaar (N=54), waren wel na twee weken beschermd. Hierbij wordt als definitie van bescherming de WHO definitie van log0.7 afname van infectieuze gele koorts 17D-virus in de plaque reductie test aangehouden, gebaseerd op een seroprotectiestudie bij non-humane primaten. (4)

Mogelijk is dus de antistofrespons de eerste twee weken na vaccinatie vertraagd bij ouderen, bijvoorbeeld door de leeftijdsafhankelijke afname van naïeve lymfocyten of toegenomen aantal of een verhoogde activiteit van T regulatoire cellen (5). Juist tijdens de periode kort na vaccinatie (tot 2 weken na vaccinatie) repliceert het vaccinvirus. (6,7) Ter illustratie is een grafiek toegevoegd die deze hypothese visualiseert (fig. 1). Bij een achterblijvende antistofrespons kan het virus meer repliceren wat leidt tot een hogere en langer aanhoudende viremie. Indien het evenwicht tussen neutraliserende antistoffen (die tot nu toe geacht worden de belangrijkste rol te spelen in bescherming tegen gele koorts) (6) en virus in het voordeel van de laatste verstoord raakt kan de vaccinatie tot ziekte leiden.

Echter, de meeste ouderen kunnen deze viremie toch beperken door een evenwicht tussen neutraliserende antistoffen en vaccinvirus te handhaven dat ten gunste is van de antistoffen, en ontwikkelen dientengevolge een goede immuunrespons die na 30 dagen gelijk is aan die van jongeren (fig. 1).

Door gele koorts 17D viremie, neutraliserende antistoffen en cellulaire immuniteit tegen gele koorts te bepalen op dezelfde tijdstippen in dezelfde personen, en deze in de groepen jonger dan 60 jaar of 60 jaar of ouder in te delen kan de invloed van leeftijd op de hoogte en de duur van de viremie, de humorale en de cellulaire respons en de invloed van deze respons op de viremie bestudeerd worden.

#
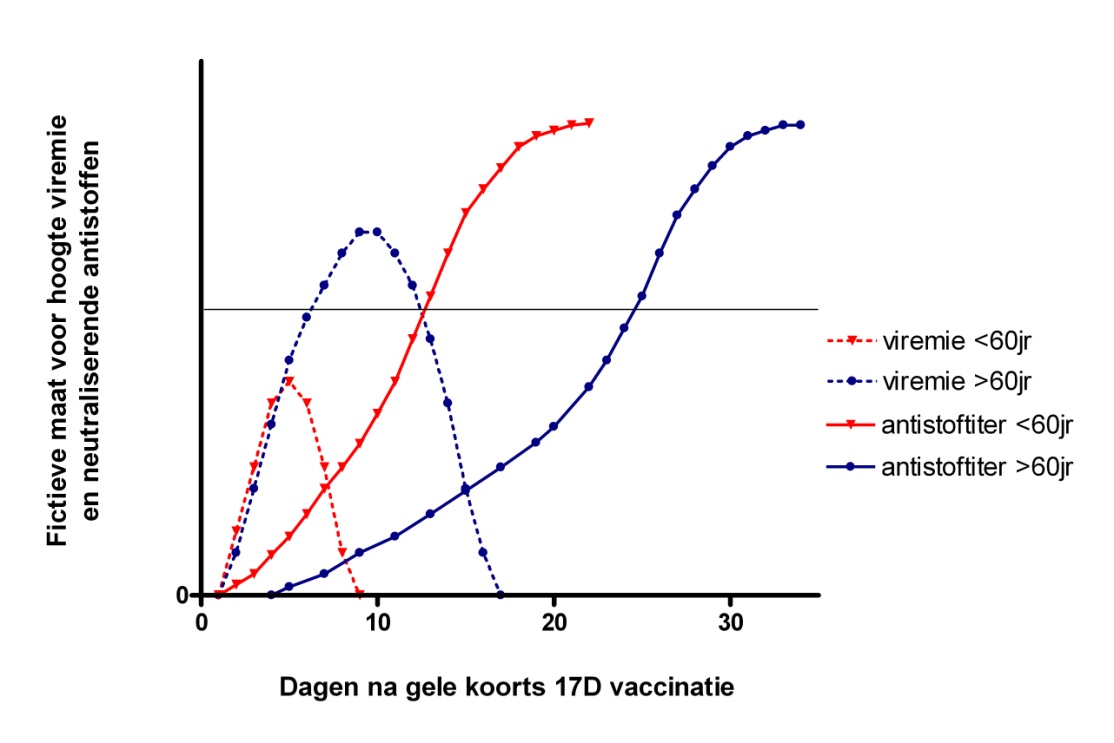


*Figuur 1. Weergave van hypothese hoe viremie en neutraliserende antistofvorming van personen boven 60 jaar (bolletjes) zich verhouden tot die van personen jonger dan 60 jaar (driehoekjes). De doorgetrokken horizontale lijn representeert de antistoftiter die beschermend is tegen de ziekte gele koorts.*

# 2. Vraagstellingen

1. Treedt de ontwikkeling van een beschermende immuunrespons (gemeten in neutraliserende antistoffen) op het gele koorts vaccinvirus bij ouderen (>60 jaar) later op dan bij jongeren?
2. Duurt de viremie bij ouderen (>60 jaar) langer en is deze hoger dan bij jongeren (<60 jaar), waarvoor een leeftijdsgroep tussen 18 en 40 jaar genomen wordt?

# 3. Doel

Het doel is het onderzoeken van het biologisch mechanisme waaraan de verhoogde kans op het optreden van gele koorts geassocieerde bijwerkingen bij ouderen (>60 jaar) ten grondslag ligt.

# 4. Onderzoeksontwerp

Het betreft een single center prospectief cohortonderzoek waarbij de immunologische respons op gele koorts vaccinatie bij reizigers van 60 jaar of ouder wordt onderzocht ten opzichte van de immunologische respons bij jongeren. De gele koorts vaccinatie die gegeven wordt in dit onderzoek wijkt niet af van de conventionele vaccinatie: een subcutane toediening van 0,5ml 17D gele koorts vaccin (Stamaril).

Aan de reizigers die aan de inclusiecriteria voldoen en op het spreekuur van de GGDs komen krijgen hier informatie over het onderzoek, waarbij zij ook de schriftelijke deelnemersinformatie ontvangen.

Indien de reiziger (via de GGD of via het LUMC) bereid is deel te nemen, wordt een afspraak gemaakt in het LUMC met de onderzoeker (AR) of onderzoeks-verpleegkundige van het LUMC. Tijdens de eerste afspraak in het LUMC wordt het toestemmingsformulier getekend, nadat de deelnemer voldoende gelegenheid heeft gekregen eventuele vragen over het onderzoek te stellen en deze naar tevredenheid zijn beantwoord. Na het geven van toestemming voor deelname wordt gevaccineerd en een bloed- en urinemonster afgenomen. De daaropvolgende bloedafnames zullen bij de deelnemer thuis plaatsvinden (tenzij deze verkiest naar het LUMC te komen).

Vaccinatie, bloed- en urinemonsterafname worden uitgevoerd door een onderzoeksverpleegkundige van het LUMC of door onderzoeker AR.

De inclusie van deelnemers zal naar verwachting 1 jaar duren. Met name de deelnemers in de ouderengroep bepalen de tijd waarin inclusie zal plaatsvinden omdat deze relatief schaars zijn.

# 5. Onderzoekspopulatie

## 5.1 Populatie

Reizigers die 60 jaar zijn of ouder en die volgens LCR protocol een indicatie hebben voor gele koorts vaccinatie op grond van een reëel infectiegevaar tijdens hun verblijf, wordt gevraagd deel te nemen aan het onderzoek (zij worden dus tegen gele koorts gevaccineerd, ongeacht deelname aan dit onderzoek). Reizigers die op het reizigersspreekuur van het LUMC en de GGD Hollands Midden en GGD Den Haag komen, en die aan de inclusiecriteria voldoen worden uitgenodigd om deel te nemen aan het onderzoek en ontvangen de deelnemersinformatie die op hen van toepassing is.

# Deelnemers voor de groep jonger dan 60 jaar worden gerekruteerd op de vaccinatiepolikliniek van het LUMC (bij indicatie voor vaccinatie), of via advertenties, (bij geen indicatie voor gele koorts vaccinatie). Deze deelnemers zijn tussen 18 t/m 40 jaar om het contrast tussen de verwachte resultaten uit de oudere en jongere groepen te vergroten.

## 5.2 Inclusiecriteria

*1.Ouderen*

*-*leeftijd > 60 jaar

-indicatie voor gele koorts vaccinatie volgens protocollen Landelijke Coördinatiecentrum Reizigersadvisering (LCR)

*2. Jongeren*

-leeftijd > 18 jaar en <40jaar

## 5.3 Exclusiecriteria

De exclusiecriteria zijn dezelfde als de contra-indicaties voor gele koorts vaccinatie die opgenomen zijn in het protocol van het Landelijk Coördinatiecentrum voor Reizigersadvisering. Dit zijn:

-Een (bekende) immuunstoornis ten gevolge van een medische conditie die de afweer ernstig vermindert zoals; maligniteit, HIV-infectie, diabetes mellitus en gebruik van immuunsuppressieve medicatie waaronder corticosteroiden en cytostatica.

-Zwangerschap

Daarnaast geldt voor dit onderzoek het volgende exclusiecriterium:

-Eerdere vaccinatie tegen gele koorts (ongeacht de tijd sinds vaccinatie). Eerder gevaccineerden kunnen een bestaande immuunrespons hebben en bij revaccinatie is geen verhoogde kans op vaccingeassocieerde ziekte waargenomen. Mogelijk wordt de immuunrespons door aanwezig immunologisch geheugen snel na vaccinatie geïnduceerd waardoor deze groep voor dit onderzoek niet geschikt is.

## 5.4 Berekening van de populatiegrootte

Bij een beschermende antistofrespons op dag 14 na vaccinatie bij een leeftijd van 18jr t/m 40jr van 95% en bij een leeftijd > 60jr van 66% zijn per groep 26 deelnemers nodig om het verschil in neutraliserende antistoffen aan te tonen, bij een α=0.05 en β=2.0. Om rekening te houden met een uitval van 15% worden per groep 30 deelnemers geϊncludeerd.

Voor berekening van de populatiegrootte is de volgende formule gebruikt: bij een α= 0.05 en β=0.2 geldt N=8x(P_1_(1-P_1_)+P_2_(1-P_2_))/(P_1_-P_2_)^2^ waarbij P_1_=95 en P_2_=66. Hierbij is N=26 (afgerond) per groep. Om rekening te houden met uitval streven naar inclusie van 30 deelnemers per groep.

Het percentage van 95% van de jongerengroep is gebaseerd op de WHO gedefinieerde effectiviteit van het vaccin en het feit dat in voorgaand (nog ongepubliceerd) onderzoek bij 100% van de deelnemers (onder 60 jaar) een beschermende respons op dag 14 werd gemeten. Het percentage van 66% is niet gebaseerd op literatuur, aangezien deze bij ons weten niet bestaat, maar op een door ons gekozen grens die wij hopen te meten waarbij 1 op 3 ouderen geen beschermende antistoftiter heeft na 14 dagen.

# 6. Behandeling van deelnemers

## 6.1 Onderzoeksproduct

Gele koorts 17D vaccin: Stamaril. Het vaccin dat gebruikt wordt bevat het levend verzwakte gele koorts 17D virus. Stamaril wordt geproduceerd door Sanofi Pasteur (zie document D2 van het onderzoeksdossier NL18398.000.07).

Met bijgeleverde injectiespuit wordt het vaccin (0,5ml) op gebruikelijke wijze (subcutaan) toegediend.

## 6.2 Co-interventies

Deelnemers mogen geen geneesmiddelen gebruiken die invloed hebben op het immuunsysteem. Geneesmiddelen die niet beogen het immuunsysteem te beïnvloeden (m.a.w. die gegeven worden met als doel de immuniteit te verhogen of te verlagen) mogen gebruikt worden tijdens het onderzoek.

Andere vaccinaties mogen gegeven worden tijdens het onderzoek, behalve andere levend verzwakte vaccins. Deze zijn gecontraindiceerd in de eerste 4 weken na gele koorts vaccinatie omdat bij sequentiële (binnen 4 weken) vaccinatie met levend verzwakte vaccins de werkzaamheid van de later gegeven vaccins verminderd kan zijn. Omdat de levend verzwakte vaccins die in Nederland worden gegeven voornamelijk kindervaccinaties zijn (BMR, BCG, varicella) en wij geen deelnemers onder 18 jaar includeren verwachten wij geen gelijktijdige vaccinatie met verschillende levend verzwakte virusvaccins.

#

#

# 7. Medicatie gebruikt in het onderzoek

Hiervoor wordt verwezen naar document D2 in het onderzoeksdossier (Investigator’s Medicinal Product Dossier 23-07-2007).

# 8. Onderzoeksmethoden

## 8.1 Eindpunten

### 8.1.1 Primaire eindpunten

Primaire eindpunten van dit onderzoek zijn

Viremie: Hoogte en duur. Dit wordt gemeten op 0, 3, 5, 10 en 14

dagen na vaccinatie

Neutraliserende antistoffen: Seroprotectie op dag 14 na vaccinatie,

kinetiek respons. Antistoffen worden

gemeten op dag 0, 3, 5, 10, 14 en 28

dagen na vaccinatie.

Cellulaire immuunrespons: Proliferatie CD4 en CD8 cellen op

stimulatie met geïnactiveerd gele koorts

virus als antigeen. Aanwezigheid T

regulatoire cellen. Cytokineprofiel bij

proliferatieassay. Cellulaire respons wordt

gemeten op 0 en 14 dagen na vaccinatie.

Bijwerkingen: Dagboekje (3 weken) waarin expliciet naar de volgende

bijwerkingen wordt gevraagd: roodheid, zwelling en pijn op de plaats van injectie, en spierpijn en koorts als systemische symptomen. Andere bijwerkingen en medicatiegebruik dienen ook te worden genoteerd

De gemeten eindpunten bij ouderen (>60jaar) worden vergeleken met de eindpunten gemeten bij jongeren (18 t/m 40 jaar).

### 8.1.2 Secundaire eindpunten

Een secundair eindpunt in deze studie is de aanwezigheid van gele koorts virus of delen van het gele koorts virus in de urine ten tijde van viremie, gecorreleerd aan de duur en hoogte van virus aanwezigheid in het bloed.

## 8.2 Studie procedures

De interventies die plaats zullen vinden zijn schematisch weergegeven in tabel 1.

Hieronder vallen: gele koorts vaccinatie (alleen een extra interventie in deelnemers uit de jongeren groep die geen indicatie tot vaccinatie hebben), venapunctie, urinemonsterafname en het bijhouden van een dagboek gedurende 3 weken (deze staat niet in de tabel).

|  | **Tijd (dagen)** | | | | | |  |
| --- | --- | --- | --- | --- | --- | --- | --- |
|  | **0** | **3** | **5** | **10** | **14** | **28** | |
| **Gele koorts vaccinatie** | x |  |  |  |  |  | |
| **Bloedafname** | x | x | x | x | x | x | |
| **Urinemonster** | x | x | x | x | x |  | |
| **Aantal buisjes (ml)** | 5 (40) | 2 (16) | 2 (16) | 2 (16) | 6 (48) | 1 (8) | |

*Tabel 1. Schema vaccinatie en bloed- en urinemonsterafname GK vaccinatie onderzoek bij 60^+^*

Testen die uitgevoerd worden om de eindpunten te onderzoeken:

Neutraliserende antistoffen: Serum voor het onderzoek naar neutraliserende antistoffen wordt afgenomen in een stolbuis. 1 buis (=8ml) volstaat voor de bepaling. Het onderzoek naar neutraliserende antistoffen, wordt uitgevoerd op het laboratorium van de afdeling Infectieziekten (LUMC) volgens de standaard procedure, te verstaan een virus neutralisatie plaque reductie test. De uitvoering van deze test staat beschreven in bijlage 1. Het serum wordt bewaard bij -20°C.

Cellulaire immuniteit: PBMCs (perifere bloed mononucleaire cellen) voor het onderzoek naar cellulaire immuniteit wordt afgenomen in een natrium-heparine buis. 5 buizen (1 buis = 8ml) volstaat voor de bepaling. Het onderzoek naar cellulaire immuniteit, wordt uitgevoerd op het laboratorium van de afdeling Infectieziekten (LUMC). Cellen worden geïsoleerd d.m.v. Ficoll-Paque scheiding (bijlage 2) en ingevroren bij -70°C en na 2 weken tot -152°C tot uitvoering van de proliferatietesten (bijlage 3).

Viremie: Plasma voor onderzoek naar gele koorts viremie wordt afgenomen in een EDTA-buis. 1 buis (=8ml) volstaat voor de bepaling. Bepaling zal plaatsvinden op het laboratorium van de afdeling Infectieziekten (LUMC) d.m.v. RT-PCR (real-time polymerase chain reaction) (bijlage 4).

Urinemonsters worden afgenomen voor meting van gele koorts vaccinvirus (geheel virus of afbraakproducten). Na afname worden de monsters ingevroren en bewaard bij -70°C tot ze getest worden m.b.v. massaspectrometrie op de afdeling parasitologie van het LUMC.

Dagboekje: Aan alle deelnemers wordt gevraagd eventuele bijwerkingen van de gele koorts vaccinatie gedurende 3 weken te noteren in een dagboekje dat na vaccinatie wordt meegegeven.

## 8.3. Terugtrekking uit het onderzoek

Deelnemers kunnen zich op ieder moment zonder opgave van reden terugtrekken uit het onderzoek. De onderzoekers kunnen besluiten deelnemers uit het onderzoek te halen wegens urgente medische redenen.

# 9. Bijwerkingenrapportage

## 9.1 Sectie 10 WMO event

In overeenkomst met sectie 10, subsectie 1, van de WMO, zal de onderzoeker de deelnemers en Toetsende Commissie informeren bij het optreden van een bijwerking die, zoals lijkt op basis van voorkomen, een veel groter nadeel met deelname aan het onderzoek met zich meebrengt dan werd verwacht bij het opzetten van het onderzoek. De studie zal opgeschort worden en voortgang zal beoordeeld worden door de Toetsende Commissie, tenzij opschorting de gezondheid van de deelnemers in gevaar brengt. De onderzoeker zal ervoor zorgen dat alle deelnemers worden geïnformeerd.

## 9.2 Bijwerkingen en ernstige bijwerkingen

Alle bijwerkingen worden gedocumenteerd door middel van een dagboekje dat gedurende drie weken na vaccinatie bijgehouden wordt.

Het gele koorts vaccin is een geregistreerd vaccin met bekende bijwerkingen (zoals spierpijn en lichte koorts) waarvoor geen aanwijzing is dat deze heviger of anders verlopen bij mensen van 60 jaar of ouder.

Bijwerkingen die kunnen optreden bij gele koorts vaccinatie zijn:

1. lokale reacties zoals roodheid en zwelling op de injectieplaats (>10%)
2. systemische reacties zoals spierpijn en lichte koorts (tussen 1-10%)

Zelden treden de volgende bijwerkingen op: allergische reacties, hoofdpijn, jeuk, huiduitslag, urticaria, geelzucht, bursitis, neuritis en lymfadenopathie.

Gele koorts vaccinatie geassocieerde ziekte kan een een neurotrope en viscerotrope uiting hebben (ernstige bijwerkingen). Bij personen van 60 jaar of ouder wordt de prevalentie van de neurotrope variant (YEL-AND) na vaccinatie geschat op 1.3/10^5^ doses en van de viscerotrope variant (YEL-AVD) geschat op 2.2/10^5^ doses. (1)

Alle ernstige bijwerkingen (SAEs = serious adverse events) worden door de onderzoeker gemeld aan de Toetsende Commissie die het protocol heeft goedgekeurd.

### 9.2.1 Suspected unexpected serious adverse reactions (SUSAR)

Wanneer een SUSAR optreedt en de onderzoeker van het desbetreffende centrum op de hoogte wordt gesteld wordt contact opgenomen met de coördinerende onderzoeker (AR) of dr. L.G. Visser die toegang hebben tot de gecodeerde deelnemerslijst. Omdat in dit onderzoek elke deelnemer de gele koorts vaccinatie krijgt (en niemand een placebo) is in principe geen decodering vereist bij een SUSAR.

De sponsor zal alle SUSARs melden aan de Toetsende Commissie, de Bevoegde Instantie, de geneesmiddelen evaluatie raad en de bevoegde instanties in andere lidstaten.

De rapportage van SUSARs zal niet later geschieden dan 15 dagen nadat de sponsor ervan op de hoogte is. Deze termijn bestaat uit 7 dagen (voorlopig rapport) en 8 dagen (definitief rapport) wanneer het een fatale of levensbedreigende bijwerking betreft.

### 9.2.2. Jaarlijkse bijwerkingen rapport

Het jaarlijkse bijwerkingen rapport wordt gecombineerd met het jaarlijkse voortgangsrapport.

## 9.3 Follow-up van bijwerkingen

Bijwerkingen worden vervolgd totdat zij verdwenen zijn of totdat zij in een stabiele toestand zijn.

# 10. Statistische analyse

Voor de vergelijking van het aantal jongeren dat de beschermende antistoftiter heeft op dag 14 na vaccinatie t.o.v. het aantal ouderen kan een Chi-kwadraat toets uitgevoerd worden. Voor vergelijking van de duur en de hoogte van viremie, evenals de hoogste serumverdunning waarbij log0.7 virus neutralisatie plaatsvindt, kan een t-toets gebruikt worden. Dit uiteraard voor het geval de populaties normaal verdeeld zijn. Bij non-parametrische verdeling gelden de non-parametrische varianten (zoals Mann-Whitney i.p.v. de t-toets). De correlatie tussen hoogte van viremie en hoogte van de antistofrespons kan geanalyseerd worden d.m.v. lineaire regressie.

Omdat onze ervaring met de cellulaire immunogeniciteits bepaling (proliferatie assay) nog niet erg ruim is, kunnen we over de analyse van de resultaten nog geen definitieve uitspraak doen. Dit geldt ook voor de virusbepaling in de urine. Naar verwachting leveren deze bepalingen kwantitatieve waarden die vergeleken kunnen worden tussen de twee groepen met eerder genoemde statistische test (t-toets).

Omdat de onderzoeksgroepen klein zijn zal geen interim-analyse plaatsvinden.

# 11. Ethische overwegingen

## 11.1 Regulation statement

Het onderzoek zal uitgevoerd worden volgens de principes van de Declaratie van Helsinki (Tokyo, 9-10-2004), en in overeenkomst met de Wet medisch-wetenschappelijk onderzoek met mensen (WMO).

## 11.2 Recrutering en toestemming

De proefpersonen van 60 jaar of ouder met een indicatie voor gele koorts vaccinatie worden geïnformeerd over het onderzoek tijdens het reizigersspreekuur op de reizigerspoliklinieken van het LUMC, GGD Hollands Midden en GGD Den Haag.

Deelnemers voor de groep jonger dan 60 jaar worden gerekruteerd tijdens het reizigersspreekuur op de reizigerspolikliniek van het LUMC en via advertenties, en hebben niet per se een indicatie voor gele koorts vaccinatie.

Aan de mogelijke deelnemers wordt schriftelijk de deelnemersinformatie verstrekt. Als zij deze informatie gelezen en begrepen hebben (waarvoor zij de tijd krijgen die ze nodig achten), en akkoord gaan met deelname, ondertekenen zij het toestemmingsformulier. Dit zal plaatsvinden tijdens de eerste afspraak op de vaccinatiepolikliniek van het LUMC, zowel voor de ouderen (≥60 jaar) en de controle (18-40) groep. Indien zij vragen hebben na het doorlezen van de deelnemersinformatie kunnen die gesteld worden aan de reizigersconsulenten, die weer kunnen doorverwijzen naar de onderzoeker in het betreffende centrum, die weer kan doorverwijzen naar de coördinerende onderzoeker A. Roukens en onderzoeker L. Visser.

## 11.3 Verantwoording voor het onderzoek en groeps-gerelateerdheid

Het onderzoek is groepsgerelateerd in de zin dat we twee leeftijdsgroepen met elkaar vergelijken. Mogelijke deelnemers moeten dus in een van de twee groepen vallen om mee te kunnen doen.

De tijdsbelasting van het onderzoek bestaat voornamelijk uit de bezoeken aan de vaccinatiepolikliniek of dat ze thuis moeten zijn op afgesproken tijdstip voor bloed- en urinemonsterafname. Verder bestaat de belasting uit de venapuncties en urinemonsterafnames.

# Onzes inziens is deze belasting verantwoord omdat de uitkomst van dit onderzoek kan een bijdrage leveren aan de opheldering van de mogelijk leeftijdsafhankelijke kinetiek van de immuunrespons geïnduceerd door gele koorts vaccinatie. Daarnaast zou dit onderzoek bijdrage kunnen leveren aan de kennis over leeftijdsafhankelijke respons op andere vaccins.

Aangezien de deelnemers de belasting van het onderzoek schriftelijk vernemen en als zij hierover vragen hebben informatie krijgen, verwachten we dat zij de belasting verantwoord zullen vinden indien zij toestemming verlenen voor deelname.

## 11.4 Vergoeding

Als tegemoetkoming voor deelname ontvangen alle deelnemers 10 euro per bloedafname. Bij afronding van het onderzoek, dus na zes bloedafnames ontvangt de deelnemer 60 euro. De deelnemers in de jongeren groep die geen indicatie hebben voor gele koorts vaccinatie worden gratis gevaccineerd.

## 11.5 Compensatie voor schade door deelname aan het onderzoek

De sponsor heeft een aansprakelijkheidsverzekering in overeenkomst met artikel 7, subsectie 6 van de WMO

De sponsor heeft ook een verzekering in overeenkomst met de juridische eisen in Nederland (artikel 7 WMO en maatregel betreffende de verplichte verzekering voor klinisch onderzoek in mensen van 23 juni 2003). Deze verzekering dekt schade opgelopen door deelnemers aan het onderzoek ten gevolge van letsel of dood veroorzaakt door het onderzoek.

1. € 450.000,= als maximum per aanspraak per proefpersoon, met een maximum van
2. € 3.500.000,= per afzonderlijk wetenschappelijk onderzoek, met dien verstande dat indien verzekerden meerdere wetenschappelijke onderzoeken verrichten of hebben verricht het totale verzekerde bedrag is gelimiteerd tot
3. € 5.000.000,= voor schade die zich per verzekeringsjaar door wetenschappe-lijk onderzoek openbaart.

# 12. Administratie en publicatie

## 12.1 Omgang en opslag medische- en onderzoeksgegevens

De medische gegevens van de proefpersonen die voor dit onderzoek door de verantwoordelijke onderzoekers verzameld worden, vallen onder het medisch beroepsgeheim. De uitkomsten van het onderzoek zullen alleen anoniem worden gepubliceerd en zijn nooit te herleiden tot de individuele proefpersonen. Behalve de onderzoeksverantwoordelijken en medewerkers aan wie door deze verantwoordelijk-en toestemming is verleend, heeft niemand inzage in de gegevens van de proefpersonen.

## 12.2 Amendementen

Alle substantiële amendementen zullen bekendgemaakt worden aan de Toetsende Commissie en de Bevoegde Instantie. Niet-susbstantiële amendementen zullen niet bekendgemaakt worden aan de Toetsende Commissie en de Bevoegde Instantie, maar worden gedocumenteerd door de sponsor.

## 12.3 Jaarlijkse rapportage

De sponsor of onderzoeker zal eens per jaar de Toetsende Commissie op de hoogte stellen van de voortgang van de studie. De volgende informatie zal hierin verstrekt worden: inclusiedatum van de eerste deelnemer, aantal deelnemers geïncludeerd, aantal deelnemers dat de studie heeft afgerond, opgetreden ernstige bijwerkingen, andere problemen die zich hebben voorgedaan en amendementen.

## 12.4 Rapportage van het einde van de studie

De sponsor of onderzoeker zal de Toetsende Commissie en Bevoegde Instantie binnen 90 dagen na beëindiging van de studie hiervan op de hoogte stellen. Het einde van de studie is gedefinieerd als het laatste bezoek van de laatste deelnemer.

Bij voortijdige beëindiging van de studie worden de Toetsende Commissie en de Bevoegde instantie hiervan binnen 15 dagen na beëindiging op de hoogte gesteld. Hierbij worden de redenen voor voortijdige beëindiging opgegeven.

Binnen 1 jaar na beëindiging van de studie zal de onderzoeker of sponsor een eindrapport overhandigen aan de Toetsende Commissie en de Bevoegde Instantie. Bij dit eindrapport horen alle publicaties en abstract die voortkomen uit het onderzoek.

## 12.5 Verslaglegging

Publicatie van de resultaten uit dit onderzoek zal volgens de volgende auteursvolgorde geschieden: A.H.E. Roukens, H. Brockhoff, C. Jobse, J.T. van Dissel, L.G. Visser. Waarbij het 2^de^ en 3^de^ auteurschap wordt ingevuld naar gelang de bijdrage van het aantal deelnemers aan het onderzoek.

# 13. Referenties

(1) Khromava AY, Eidex RB, Weld LH, Kohl KS, Bradshaw RD, Chen RT et al. Yellow fever vaccine: an updated assessment of advanced age as a risk factor for serious adverse events. Vaccine 2005; 23(25):3256-3263.

(2) Martin M, Tsai TF, Cropp B, Chang GJ, Holmes DA, Tseng J et al. Fever and multisystem organ failure associated with 17D-204 yellow fever vaccination: a report of four cases. Lancet 2001; 358(9276):98-104.

(3) Monath TP, Cetron MS, McCarthy K, Nichols R, Archambault WT, Weld L et al. Yellow fever 17D vaccine safety and immunogenicity in the elderly. Hum Vaccin 2005; 1(5):207-214.

(4) Mason RA, Tauraso NM, Spertzel RO, Ginn RK. Yellow fever vaccine: direct challenge of monkeys given graded doses of 17D vaccine. *Appl Microbiol* 1973(4); 25: 539−544.

(5) Pawelec G. Immunity and ageing in man. Exp Gerontol. 2006; 41(12):1239-42

(6) Monath TP. Yellow Fever. In: Plotkin SA, Orenstein WA, editors. Vaccines. W.B. Saunders Company 1999; 815−879.

(7) Reinhardt B, Jaspert R, Niedrig M, Kostner C, L'age-Stehr J. Development of viremia and humoral and cellular parameters of immune activation after vaccination with yellow fever virus strain 17D: a model of human flavivirus infection. J Med Virol 1998; 56: 159−167.

(8) World Health Organization Expert Committee on Biological Standardization. 46^th^ report. WHO Technical Report ser. No. 872. Geneva, World Health Organization 1998.

# Bijlage 1

Protocol Plaque Reductie Virus Neutralisatie test

1. 6-wells platen: monolayer Verocellen kweken (één 175 cm2 kweekfles voor 6-8 platen)
2. gele koorts vaccin virus (stock concentratie 3.10^6^ PFU (plaque forming units)/ml (opslag -70°C, linker vriezer, toren Anna, rechts)
3. serum (pre- (x.1) en postvaccinatie (x.2, x.3 en x.4)
4. proeven op ML-II lab met levend (verzwakt) virus! Filtertips gebruiken, puntjes altijd met alcohol spoelen voor weggooien
5. YFV verdunnen tot 10^3^ PFU/ml → 20μl stock in 580μl FCS 1% (10^5^ PFU/ml), daarvan 200μl in 19800ml FCS 1% (10^3^ PFU/ml)
6. Sera x.2, x.3 en x.4 verdunnen van 1:16 t/m 1:512 (2-voudige verdunningen) → 40μl serum + 600μl FCS 1%. In volgende verdunningen 320μl FCS 1% pipetteren. Steeds 320μl van verdunning 1 in 2, 2 in 3 enz.
7. Serum x.1 1 keer verdunnen (1:16 of 20μl serum in 300μl FCS 1%)
8. 250μl serumverdunning + 250μl van 10^3^ PFU/ml,‘mix gently’
9. Serum x.1 in epje 50PFU 125μl van 10^3^ PFU/ml (controle voor PFU)
10. PFU controle: 250μl virusstockverdunning + 250μl FCS 1%
11. 60’ incubatie op ijs
12. platen wassen in 1ml PBS/well (begin op tijd voor eind ijs-incubatie), en markeren (1 plaat = 3 verdunningen)
13. 200μl van virus-serum mix per well (2 wells per serumverdunning)
14. 60’ incubatie bij 37°C, af en toe platen zwenken zodat cellen niet droog staan
15. Overlay maken: 2% agarose, opwarmen in autoclaaf. 2xDMEM (2%FCS, 1% Pen/Strep) opwarmen in 43.5°C. Verdeel in meer kleine flesjes (anders stolt de overlay tijdens het aanbrengen). Zelfde hoeveelheid DMEM^++^ als agarose.
16. 5 dagen incuberen bij 37°C, dag na inzetten platen omdraaien
17. Cellen fixeren: 7% formaldehyde, 2.5ml/well, 30-60min incubatie kamertemperatuur (zuurkast!)
18. Formaldehyde verwijderen, overlay met spateltje verwijderen (zuurkast! overlay in apart afvalzakje), aankleuren met cristalviolet

Benodigde oplossingen:

- 1%FCS = 5ml FCS in 500ml PBS (bewaren in 4°C)

- 2% agarose = 2g agarose per 100ml milliQ (autoclaveren en afkoelen tot 43,5°C)

- Medium voor celkweek: DMEM (500ml + 5ml Pen/Strep + 5ml glutamax + 50ml FCS)

- DMEM++ voor overlay (apart protocol, 5 liter per keer maken)

- 7% formaldehyde = standaardoplossing 35% dus 100ml formaldehyde + 400ml water (uit de kraan) Oplossingen bewaren bij 20°C

- Cristalviolet = 2,5g Cristalviolet oplossen in 100ml 40% ethanol. Werkoplossing maken: 50ml van CV oplossing + 50ml H_2_O

# Bijlage 2

Protocol PBMC-isolatie en proliferatieassay

PBMC isolatie uit volbloed:

1. Put the code number of the sample on two 50 mL tubes and on the lids.
2. Fill per sample a 15 ml tube with 10 ml of sterile PBS (using a pipette).
3. Make sure the lid of the heparin tube is closed well. Turn heparin tube up and down several times to mix remaining plasma and blood cells.
4. Open the tube, be careful to put the lid upside down in the cabinet
5. Pipette the blood from the heparin tube into a 50 ml tube (Ficoll tube),
6. Next, using the same pipette aspirate ca 10 mL of sterile PBS from the 15 ml tube to rinse the blood from the pipette. Add this PBS to the Ficoll tube.
7. Add PBS to the Ficoll tube until 25 ml.
8. Bring 10 ml Ficoll under the blood.
9. Centrifuge the tube at 1800 rpm for 20 minutes brake off (this will take about 30 minutes).
10. Harvest the PBMC ring by using a Pasteur pipette. Put the PBMCs in a new 50 ml tube; the washing tube.
11. Fill the washing tube to 45 ml with PBS.
12. Centrifuge at 1800 rpm for 10 minutes at room temperature with the brake on.
13. Pour the above standing fluid into the white carton box. Resuspend the pellet.
14. Fill the washing tube with PBS until 12.5 ml.
15. Centrifuge at 1200 rpm for 10 minutes at room temperature with brake on.
16. Repeat step 15
17. Centrifuge at 1000 rpm for 10 minutes at room temperature with brake on.

# Bijlage 3

Proliferatieassay met geïnactiveerd gele koorts 17-D virus

Day 0:

1. thaw cells in 1 ml FCS + 1 ml RPMI
2. spin 7’ at 1400 rpm
3. resuspend cells in PBS/ 0.5% BSA
4. count cells using eosin
5. spin 7’ at 1400 rpm
6. resuspend at 10x10e6/ml in PBS/ 0.5% BSA (1 ml = minimum volume)
7. keep some cells as unlabeled control
8. add CFSE (Molecular Probes C1157, stock 5mM) at concentration of 5 μM (1/1000), incubate 10 minutes at 37C (not longer)
9. add 100 μl FCS per ml of cells, add 6 ml PBS/ 0.5% BSA and spin 7’ at 1400 rpm
10. resuspend cells in IMDM + 10% Human serum
11. count cells using eosin
12. adjust cell concentration to 1.5x10e6, 100 μl/well: 150.000 cells, all conditions should be done in triplicate
13. dilute yellow fever virus to dilutions of interest and inactivate for 1 hour at 56°C
14. add inactivated YF in PFU to be tested (variating from 10^1^-10^5^) in 100 μl (2 times the final concentration). Final concentration mitogen: PHA 1/200. Always include unstimulated control (medium)

Day 7:

1. collect supernatants 100-150 μl/ well, pool triplicates
2. harvest cells, pool triplicates
3. wash with PBS/ 0.1% BSA, spin 7’ at 1400 rpm
4. add antibodies in total volume of 50 μl, CD4-PerCP 1/60, CD8-APC 1/300, incubate for 30 minutes in the fridge
5. wash with PBS/ 0.1% BSA, spin 7’ at 1400 rpm
6. FACS

# Bijlage 4

Protocol RT-PCR op gele koorts virus RNA

1. viral RNA isolation with total nucleic acid isolation kit by Magna Pure LC Instrument
2. reverse transcription of YF-17D RNA by MutiScribe reverse transcriptase + random hexamers
3. 20 µl of eluted RNA + 5 µl of 10 x RT-buffer + 5.5 mM MgCl_2_ + 500 µM concentration of each of deoxynycleoside triphosphates + a 2.5 µM concentration of random hexamer + 62.5 U of MultiScribe RT + 20 U of RNase inhibitor.
4. cDNA synthesis: annealing 10’ (25°C), reverse transcription 30’ (48°C), reverse transcriptase inactivation 5’ (95°C).
5. following YF specific primers and probe used for real-time PCR:

YFV-1 (forward) AATCGAGTTGCTAGGCAATAAACAC

YFV-2 (reverse) TCCCTGAGCTTTACCAGA

YFV-P (probe) FAM-ATCGTTGAGCGATTAGCAG-BHQ

1. 25 µl reaction mixture: 10 µl of cDNA + 12.5 µl of 2x Taqman Universal PCR Mastermix + 7.5 pmol of each of primers + 2.5 pmol of probe
2. PCR mixture 2’ at 50°C, than 10’ at 95°C (activation of AmpliTaq Gold DNA polymerase).
3. 45 cycles are performed
